# A protocol of a systematic review on deep brain stimulation surgery and its efficacy in addressing substance abuse addiction

**DOI:** 10.1002/hsr2.1409

**Published:** 2023-07-08

**Authors:** David Zammit Dimech, Redoy Ranjan

**Affiliations:** ^1^ Department of Surgical Sciences Surgical Sciences Programme, University of Edinburgh Edinburgh UK; ^2^ Department of Surgery Faculty of Surgery, Bangabandhu Sheikh Mujib Medical University Dhaka Bangladesh

**Keywords:** addiction, deep brain stimulation (DBS), substance use disorder

## Abstract

**Background:**

Pharmacotherapy and psychotherapeutic approaches are still the mainstay first line of treatment for substance use disorder. However, the path to rehabilitation and cessation of dependence often proves uncertain and laborious, with risks of relapse being considerable despite exposure to current therapeutic modalities. For cases of treatment‐refractory addiction, deep brain stimulation (DBS) interventions can prove a more effective long term therapeutic solution for the patient.

**Objectives:**

The aim of the study will be to systematically assess whether attempts at correcting substance use disorder via DBS neurosurgical interventions have been successful in inducing remission or ameliorating relapse rates.

**Methods:**

The current study will analyze available literature from database inception up to 15th April 2023, reviewing all publications documenting results achieved with human patients undergoing DBS for substance use disorder in PubMed, Ovid, Cochrane, and Web of Science. The electronic database search will exclude animal studies in the field and focus solely on the application of DBS for the purposes of addressing addiction disorders.

**Results:**

The expectation is for a reduced number of trial results to have been reported, namely due to the relatively recent application of DBS to address severe addiction. Nonetheless, numbers should be in sufficient amount to inform about the efficacy of the intervention.

**Conclusion:**

This study will attempt to demonstrate the viability of DBS as a solution for tackling treatment‐refractory substance use disorder, proposing it as a valid therapeutic option that can deliver robust results and help combat an expanding societal plague that is drug dependence.

## 1 INTRODUCTION

The potential of deep brain stimulation (DBS) surgery was first explored in the late 1980s in patients presenting with movement disorder symptomatology, for such conditions as Parkinson's Disease, dystonia and essential tremor.^1^ It was later incorporated in the treatment of neuropsychiatric pathologies, including Tourette's Syndrome, obsessive compulsive disorder, mood and eating disorders.^2^ Nonetheless, the therapeutic possibilities of electrical stimulation of the brain had been explored at least since the 1930s in ablation interventions intended to provide resolution for seizures in epilepsy.^3^

There have been rapid advances in the past decade within this branch of functional neurosurgery, to the extent that it is now possible to implant electrodes in targeted areas in the brain, such as the nucleus accumbens and the subthalamic nucleus.^4^ The intent is to modulate neural pathways in the mesolimbic and mesocortical dopaminergic system rather than ablating them, thus controlling the dependence on substances of abuse which debilitate the lives of many patients and their significant others, when cases are severe and multiple non‐DBS attempts have failed.^5^

Addiction entails a chronically relapsing disorder with presentation of compulsions for seeking and consuming substances of abuse, that despite triggering repercussions still persist in time.^6^ Craving the substance is due to neural circuitry changes that result in a marked difficulty to suppress the urge to seek the substance at the basis of the dependence.^7^ Risk of relapse in substance abuse patients is currently high when addressed with conventional pharmacotherapeutic methods. For alcoholics the rates reach around 70% within the first year of therapy and for opioid addicts it can reach up to 91%.^8^ Socioeconomic costs are significant and they increase exponentially over time as interrelated complications present a cumulatively detrimental effect. The risk of the individual being alienated from social support structures and potential financial ruin is higher as the patient gets entangled in physiological reward circuitry processes over which control only grows more ephemeral.^9^

This protocol is intended to lay groundwork and prospects for a systematic review that will observe the efficacy of DBS neurosurgical interventions in substance use disorders.

## 2 RESEARCH HYPOTHESIS

Do DBS neurosurgical interventions contribute to effectively reduce or even extinguish relapse episodes in substance use disorder patients presenting treatment‐refractory addiction pathology?

### 2.1 Research gap

Researchers observed significant results in correcting dependence on substance abuse via DBS neurosurgical interventions. Applications of DBS for treating patients with severe addictions is still in its infancy but a handful of cases have been reported, and new trials keep getting published as the field of neurosurgery seeks to offer a robust therapeutic solution. This study looks into providing an update of what results have been achieved so far with the patient population, covering a research gap that concerns knowledge about this area of functional neurosurgery and its effectiveness in dealing with severe addiction. We characterize the research gap with the PICO framework as follows:

Population (P): Substance use disorder patients admitted for DBS surgery.

Intervention (I): DBS neurosurgical intervention to treat addiction.

Comparison (C): Substance use disorder patients ahead of DBS when treated conservatively.

Outcomes (O): Improved outcome with DBS surgery.

### 2.2 Conceptual framework

The relevance of this project is the need for an updated compilation of reported patient cases to have better insight into the authenticated efficacy of the intervention and hence the possibility of more widespread DBS use in scenarios of severe addiction and frequent relapses to bolster traditional pharmaco‐psychiatric modalities of treatment.

### 2.3 Patients and methodology

This systematic review conducted in accordance with the Preferred Reporting Items for Systematic reviews and Meta‐Analyses (PRISMA) 2020 guidelines.^10^

**Search strategy**: The systematic review is concerned with the neurosurgical intervention of DBS as deployed in addressing substance dependence. The search terms employed in mining for data therefore focused on the intervention of DBS, its effectiveness as an outcome, as well as the problem defined for the patient population—addiction to different substances of abuse. The databases consulted were PubMed, Embase through Ovid, Cochrane and Web Of Science and searched for all published original research articles in English from January 1946 to April 15, 2023. Current duplicates were removed. Before finalizing the review in later stages of the study the search protocol will be re‐run for more updated search results and any new publications included, revising the current search strategy outcome. Two independent researchers reviewed the relevant studies, and in case of any conflict or disagreements, disputes are resolved, either through comparison and discussion or the engagement of a third researcher with expertise in systematic reviews.

The keywords that served as basis for the search protocol were “deep brain stimulation” and “addiction” as well as keywords referring to the various potential substances of abuse under investigation, which included the terms “heroin,” “opioid,” “smoking,” “nicotine,” “alcohol,” “benzodiazepines,” “methamphetamines,” and “cocaine.” For PubMed the following related MeSH terms were retrieved and employed alongside specific search words derived from the aforementioned keywords: “Deep Brain Stimulation”[Mesh], “Behavior, Addictive”[Mesh], “Substance‐Related Disorders”[Mesh], “Opioid‐Related Disorders”[Mesh], “Cocaine‐Related Disorders”[Mesh], “Amphetamine Related Disorders”[Mesh], “Tobacco Use Disorder”[Mesh], “Morphine Dependence”[Mesh], “Heroin Dependence”[Mesh], “Alcoholism”[Mesh], “Opium Dependence”[Mesh, “Narcotic‐Related Disorders”[Mesh]. The equivalent MeSH terms utilized in Cochrane were: “Deep Brain Stimulation,” “Behavior,” “Addictive,” “Narcotic‐Related Disorders,” “Substance‐Related Disorders,” “Opioid‐Related Disorders,” “Alcohol‐Related Disorders,” “Tobacco Use Disorder,” “Methamphetamine,” “Cocaine‐Related Disorders,” and “Benzodiazepine‐Related Disorders.” For the Ovid Embase database search the following subject headings were used alongside relevant search terms: “brain depth stimulation/,” “addiction/,” “abuse/,” “alcohol/,” “smoking/,” “nicotine/,” “tobacco/,” “opiate/,” “diamorphine/,” “benzodiazepine/,” “methamphetamine/,” and “cocaine/.”

The final study results of retrieved publications will be presented in appropriate tables, highlighting major characteristics and relevant outcomes of each study, for adequate compilation or details for review and satisfactory comparison. All participants in the studies will be required to have demonstrated problematic dependence on a substance for which they would be subjected to DBS for. This should be for a period of at least 1 year, with patients having been diagnosed as suffering from a dependence disorder. Having undertaken previous treatment attempts to address the addiction disorder will not be mandatory, as this will also depend on the specific substance.

### 2.4 Study inclusion and exclusion criteria

**Figure 1:**
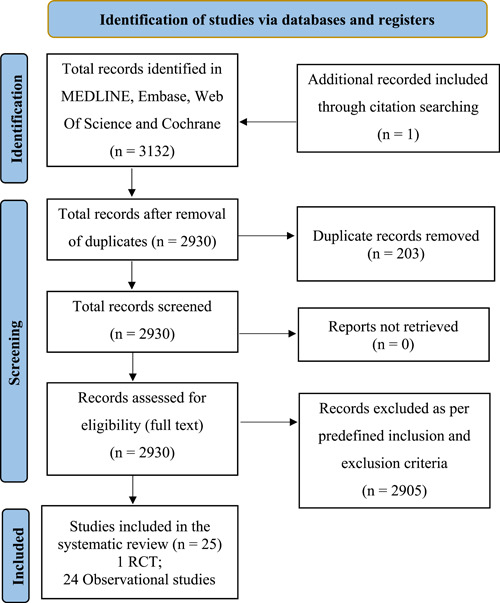
Preferred Reporting Items for Systematic Reviews and Meta‐Analyses flow diagram according to 2020 guidelines^10^ showing phases of our study.

We searched all published original research articles in English up to April 15, 2023 and identified 3132 published articles, of which 25 studies^9^, ^11^, ^12^, ^13^, ^14^, ^15^, ^16^, ^17^, ^18^, ^19^, ^20^, ^21^, ^22^, ^23^, ^24^, ^25^, ^26^, ^27^, ^28^, ^29^, ^30^, ^31^, ^32^, ^33^, ^34^ (1 Randomized Controlled Trial and 24 observational studies) met our study inclusion criteria (Figure 1). Our retrieved records were assessed in accordance with the inclusion and exclusion criteria described below. Studies were reviewed as per initial exclusion parameters, removing any publications not in English. Any analyses not concerning DBS as an intervention for substance addiction disorders were excluded. As were DBS studies focused on neuropsychiatric disorders unrelated to substance of abuse, such as eating disorders and mood disorders, or research concerning neurodegenerative disorders, including Parkinson's Disease, dystonia and epilepsy. Animal studies and literature reviews were also excluded. Duplicates were then removed from the list.

With regards to inclusion criteria the human subjects could be of any age, sex and ethnicity, and the substance referred to in the studies could be any that could lead to dependence. This while observing exclusion criteria mentioned above. The studies had to focus on addiction interventions with DBS being applied on human subjects to address substance abuse disorders.

### 2.5 Risk of bias analysis

Two independent investigators will evaluate each study for risk of bias using the Cochrane Risk of Bias tool 2.0 (ROB 2) for randomized trials^35^ and the Risk of Bias in Nonrandomized Studies (ROBINS‐I) tool for observational cohorts.^36^

## 3 LITERATURE REVIEW

Substances of abuse can generate significant distress in an individual's life when dependence develops and there is a manifest inability to control consumption. The emergence of deleterious behaviors of addiction makes it progressively more difficult to suspend use of the substance as neural reward pathways are engaged.^37^ Patterns of addiction become engrained in the individual's demeanour, and depending on the severity of the addiction and the substance itself, this can intrude heavily on daily activity and function.^38^ The potential disruption to the lives of significant others and the economic impact on society when attempting to manage chronic cases of heavy dependence and drug abuse can be high.^39^

The most frequently abused substance globally is alcohol, followed by opioids.^40^ Illicit drug use, which comprises opioids, cocaine, cannabis and amphetamines, is directly or indirectly involved in the demise of around 600,000 persons every year.^41^ It is estimated that around 2015, the potent synthetic opioid fentanyl and its analogues had made a significant impact in the illicit US drug market, with exponential growth year on year.^42^ Detected also in cocaine, heroin and methamphetamine supplies since then,^43^ it has contributed to a higher number of overdose deaths—a sixfold increase from 2015 to 2020, with 56,516 directly attributable to fentanyl alone in 2020 in the United States.^44^ Worldwide, the burden of disease and injury attributable to the use of alcohol is 5.1%. Deaths resulting from alcohol misuse amount to 5.3% globally, with 13.5% of all deaths for younger individuals within the 20–39 age bracket being due to alcohol.^45^ Nicotine is estimated to cause 7.7 million deaths yearly, with 20% of all male deaths worldwide being attributable to cigarette smoking. The number of smokers has increased to 1.1 billion people, and it is crucial to note that 9 in 10 of all smokers are considered to be addicted by 25 years of age.^41^

The mesolimbic dopaminergic system in the brain is part of the reward pathway that when subjected to unregulated processes leads to the creation and maintenance of addictive behaviors. Via the release of dopamine into the nucleus accumbens the pleasure cycle is regulated over the wanting or desire phase and the consummatory “liking” phase, as well as the reinforcement process generating learnt cues associated with the reward stimulus.^46^ Therapeutic management of some psychiatric and movement disorders have now incorporated DBS in their treatment plan due to the unrivaled benefits this neurosurgical technique has offered to patients.^47^ DBS interventions on the brain's reward system, targeting the nucleus accumbens primarily, hold promise for similarly successful results, presenting a therapeutic option for cases of severe addiction where conservative attempts have failed repeatedly.^48^

### 3.1 Novelty of the study

The research will contribute improved dissemination of knowledge related to this relatively young field offering new therapeutic possibilities for patients who fail to overcome relapse and plunge back into a vicious cycle of despair that can significantly dent the psychosocial and economic resources of the individual, with ensuing long term societal ramifications.

### 3.2 Concluding remarks

Few surgical fields can claim to be as fresh and innovative as DBS neurosurgery, especially with recent technological advancement and increasing knowledge of various neuronal networks in the brain. The prospect of being able to assist with addressing the ever‐increasing epidemic of drug addiction on a global scale effectively and tangibly, where more conservative interventions have repeatedly failed, carries hope for many patients and their relatives.

## AUTHOR CONTRIBUTIONS

**David Zammit Dimech**: Conceptualization; Data curation; Investigation; Methodology; Resources; Visualization; Writing—original draft; Writing—review & editing. **Redoy Ranjan**: Conceptualization; Data curation; Methodology; Resources; Supervision; Writing—review & editing.

## CONFLICT OF INTEREST STATEMENT

Redoy Ranjan is an Editorial Board member of Health Science Reports and a co‐author of this article. To minimize bias, he was excluded from all editorial decision‐making related to the acceptance of this article for publication. The other author declares no conflict of interest.

## TRANSPARENCY STATEMENT

The lead author Redoy Ranjan affirms that this manuscript is an honest, accurate, and transparent account of the study being reported; that no important aspects of the study have been omitted; and that any discrepancies from the study as planned (and, if relevant, registered) have been explained.

## Data Availability

This is an ongoing project and the data that support the findings of this study are available on request from the corresponding author. The data are not publicly available due to privacy or ethical restrictions. Dr. David Zammit Dimech had full access to all of the data in this study and took complete responsibility for the integrity of the data and the accuracy of the data analysis.

## References

[1] Malek N . Deep brain stimulation in parkinson's disease. Neurol India. 2019;67(4):968‐978. 31512617 10.4103/0028-3886.266268

[2] Sullivan CRP , Olsen S , Widge AS . Deep brain stimulation for psychiatric disorders: from focal brain targets to cognitive networks. Neuroimage. 2021;225:117515. 33137473 10.1016/j.neuroimage.2020.117515 PMC7802517

[3] Gardner J . A history of deep brain stimulation: technological innovation and the role of clinical assessment tools. Soc Stud Sci. 2013;43(5):707‐728.

[4] Lee DJ , Lozano CS , Dallapiazza RF , Lozano AM . Current and future directions of deep brain stimulation for neurological and psychiatric disorders. J Neurosurg. 2019;131(2):333‐342. 31370011 10.3171/2019.4.JNS181761

[5] Settell ML , Testini P , Cho S , et al. Functional circuitry effect of ventral tegmental area deep brain stimulation: imaging and neurochemical evidence of mesocortical and mesolimbic pathway modulation. Front Neurosci. 2017;11:104. 28316564 10.3389/fnins.2017.00104 PMC5334355

[6] Qu L , Ge S , Li N , et al. Clinical evaluation of deep brain stimulation of nucleus accumbens/anterior limb of internal capsule for opioid relapse prevention: protocol of a multicentre, prospective and double‐blinded study. BMJ Open. 2019;9(2):e023516. 10.1136/bmjopen-2018-023516 PMC6398661 30765398

[7] Anton RF . What is craving? Alcohol research & health: the journal of the National Institute on Alcohol Abuse and Alcoholism. 1999;23(3):165‐173. 10890811 PMC6760371

[8] Kadam M , Sinha A , Nimkar S , Matcheswalla Y , De Sousa A . A comparative study of factors associated with relapse in alcohol dependence and opioid dependence. Indian J Psychol Med. 2017;39(5):627‐633. 29200559 10.4103/IJPSYM.IJPSYM_356_17 PMC5688890

[9] Davidson B , Giacobbe P , George TP , et al. Deep brain stimulation of the nucleus accumbens in the treatment of severe alcohol use disorder: a phase I pilot trial. Mol Psychiatry. 2022;27(10):3992‐4000. 35858989 10.1038/s41380-022-01677-6

[10] Page MJ , McKenzie JE , Bossuyt PM , et al. The PRISMA 2020 statement: an updated guideline for reporting systematic reviews. BMJ. 2021;372:n71. 33782057 10.1136/bmj.n71 PMC8005924

[11] Bach P , Luderer M , Müller UJ , et al. Deep brain stimulation of the nucleus accumbens in treatment‐resistant alcohol use disorder: a double‐blind randomized controlled multi‐center trial. Transl Psychiatry. 2023;13(1):49. 36755017 10.1038/s41398-023-02337-1 PMC9908935

[12] Chen L , Li N , Ge S , et al. Long‐term results after deep brain stimulation of nucleus accumbens and the anterior limb of the internal capsule for preventing heroin relapse: an open‐label pilot study. Brain Stimulation. 2019;12(1):175‐183. 30245163 10.1016/j.brs.2018.09.006

[13] Ge S , Geng X , Wang X , et al. Oscillatory local field potentials of the nucleus accumbens and the anterior limb of the internal capsule in heroin addicts. Clin Neurophysiol. 2018;129(6):1242‐1253. 29674090 10.1016/j.clinph.2018.03.008

[14] Ge S , Chen Y , Li N , et al. Deep brain stimulation of nucleus accumbens for methamphetamine addiction: two case reports. World Neurosurgery. 2019;122:512‐517. 30448569 10.1016/j.wneu.2018.11.056

[15] Gonçalves‐Ferreira A , do Couto FS , Rainha Campos A , Lucas Neto LP , Gonçalves‐Ferreira D , Teixeira J . Deep brain stimulation for refractory cocaine dependence. Biol Psychiatry. 2016;79(11):e87‐e89. 26235303 10.1016/j.biopsych.2015.06.023

[16] Heldmann M , Berding G , Voges J , et al. Deep brain stimulation of nucleus accumbens region in alcoholism affects reward processing. PLoS One. 2012;7(5):e36572. 22629317 10.1371/journal.pone.0036572 PMC3358316

[17] Kuhn J , Lenartz D , Huff W , et al. Remission of alcohol dependency following deep brain stimulation of the nucleus accumbens: valuable therapeutic implications? J Neurol Neurosurg Psychiatry. 2007;78(10):1152‐1153. 17878197 10.1136/jnnp.2006.113092 PMC2117573

[18] Kuhn J , Bauer R , Pohl S , et al. Observations on unaided smoking cessation after deep brain stimulation of the nucleus accumbens. Eur Addict Res. 2009;15(4):196‐201. 19622886 10.1159/000228930

[19] Kuhn J , Gründler TOJ , Bauer R , et al. Successful deep brain stimulation of the nucleus accumbens in severe alcohol dependence is associated with changed performance monitoring. Addict Biol. 2011;16(4):620‐623. 21762290 10.1111/j.1369-1600.2011.00337.x

[20] Kuhn J , Möller M , Treppmann JF , et al. Deep brain stimulation of the nucleus accumbens and its usefulness in severe opioid addiction. Mol Psychiatry. 2014;19(2):145‐146. 10.1038/mp.2012.196 23337942

[21] Leong SL , Glue P , Manning P , et al. Anterior cingulate cortex implants for alcohol addiction: a feasibility study. Neurotherapeutics. 2020;17(3):1287‐1299. 32323203 10.1007/s13311-020-00851-4 PMC7641294

[22] Mahoney JJ , Haut MW , Hodder SL , et al. Deep brain stimulation of the nucleus accumbens/ventral capsule for severe and intractable opioid and benzodiazepine use disorder. Exp Clin Psychopharmacol. 2021;29(2):210‐215. 34043402 10.1037/pha0000453 PMC8422285

[23] Mantione M , van de Brink W , Schuurman PR , Denys D . Smoking cessation and weight loss after chronic deep brain stimulation of the nucleus accumbens: therapeutic and research implications: case report. Neurosurgery. 2010;66(1):E218. 20023526 10.1227/01.NEU.0000360570.40339.64

[24] Müller UJ , Sturm V , Voges J , et al. Successful treatment of chronic resistant alcoholism by deep brain stimulation of nucleus accumbens: first experience with three cases. Pharmacopsychiatry. 2009;42(6):288‐291. 19924591 10.1055/s-0029-1233489

[25] Müller U , Sturm V , Voges J , et al. Nucleus accumbens deep brain stimulation for alcohol addiction ‐ safety and clinical long‐term results of a pilot trial. Pharmacopsychiatry. 2016;49(4):170‐173. 27145161 10.1055/s-0042-104507

[26] Valencia‐Alfonso CE , Luigjes J , Smolders R , et al. Effective deep brain stimulation in heroin addiction: a case report with complementary intracranial electroencephalogram. Biol Psychiatry. 2012;71(8):e35‐e37. 22281120 10.1016/j.biopsych.2011.12.013

[27] Voges J , Müller U , Bogerts B , Münte T , Heinze HJ . Deep brain stimulation surgery for alcohol addiction. World Neurosurgery. 2013;80(3‐4):S28.e21‐S28.e31. 10.1016/j.wneu.2012.07.011 22824557

[28] Witjas T , Baunez C , Henry JM , et al. Addiction in Parkinson's disease: impact of subthalamic nucleus deep brain stimulation. Mov Disorders. 2005;20(8):1052‐1055. 10.1002/mds.20501 15858803

[29] Xu J , Wang G Therapeutic effect of deep brain stimulation of the nucleus accumbens on refractory drug addiction: a case report. Poster presented at: Annual International Neuromodulation Society; December 9‐12, 2007; Acapulco, Mexico.

[30] Zhang C , Huang Y , Zheng F , Zeljic K , Pan J , Sun B . Death from opioid overdose after deep brain stimulation: a case report. Biol Psychiatry. 2018;83(1):e9‐e10. 28882316 10.1016/j.biopsych.2017.07.018

[31] Zhang C , Wei H , Zhang Y , et al. Increased dopamine transporter levels following nucleus accumbens deep brain stimulation in methamphetamine use disorder: a case report. Brain Stimulation. 2019;12(4):1055‐1057. 30853339 10.1016/j.brs.2019.02.023

[32] Zhang C , Li J , Li D , Sun B . Deep brain stimulation removal after successful treatment for heroin addiction. Aust N Z J Psychiatry. 2020;54(5):543‐544. 31782321 10.1177/0004867419890671

[33] Zhou H , Xu J , Jiang J . Deep brain stimulation of nucleus accumbens on heroin‐seeking behaviors: a case report. Biol Psychiatry. 2011;69(11):e41‐e42. 21489407 10.1016/j.biopsych.2011.02.012

[34] Zhu R , Zhang Y , Wang T , et al. Deep brain stimulation of nucleus accumbens with anterior capsulotomy for drug addiction: a case report. Stereotact Funct Neurosurg. 2020;98(5):345‐349. 32846423 10.1159/000509313

[35] Sterne JAC , Savović J , Page MJ , et al. RoB 2: a revised tool for assessing risk of bias in randomised trials. BMJ. 2019;366:l4898. 31462531 10.1136/bmj.l4898

[36] Sterne JA , Hernán MA , Reeves BC , et al. ROBINS‐I: a tool for assessing risk of bias in non‐randomised studies of interventions. BMJ. 2016;355:i4919. 27733354 10.1136/bmj.i4919 PMC5062054

[37] Volkow ND , Morales M . The brain on drugs: from reward to addiction. Cell. 2015;162(4):712‐725. 26276628 10.1016/j.cell.2015.07.046

[38] Volkow ND , Michaelides M , Baler R . The neuroscience of drug reward and addiction. Physiol Rev. 2019;99(4):2115‐2140. 31507244 10.1152/physrev.00014.2018 PMC6890985

[39] Rehm J , Shield KD . Global burden of disease and the impact of mental and addictive disorders. Curr Psychiatry Rep. 2019;21(2):10. 30729322 10.1007/s11920-019-0997-0

[40] Degenhardt L , Charlson F , Ferrari A , et al. The global burden of disease attributable to alcohol and drug use in 195 countries and territories, 1990–2016: a systematic analysis for the global burden of disease study 2016. The Lancet Psychiatry. 2018;5(12):987‐1012. 30392731 10.1016/S2215-0366(18)30337-7 PMC6251968

[41] Global Burden of Disease Collaborative Network . Global Burden of Disease Study 2019 (GBD 2019) Smoking Tobacco Use Prevalence 1990‐2019. Institute for Health Metrics and Evaluation (IHME); 2021.

[42] Tuazon E , Kunins HV , Allen B , Paone D . Examining opioid‐involved overdose mortality trends prior to fentanyl: New York City, 2000‐2015. Drug Alcohol Depend. 2019;205:107614. 31689642 10.1016/j.drugalcdep.2019.107614

[43] Han Y , Yan W , Zheng Y , Khan MZ , Yuan K , Lu L . The rising crisis of illicit fentanyl use, overdose, and potential therapeutic strategies. Transl Psychiatry. 2019;9(1):282. 31712552 10.1038/s41398-019-0625-0 PMC6848196

[44] National Institutes of Health . Overdose Death Rates. National Institute on Drug Abuse. Published January 20 2022. Accessed January 18 2023. https://nida.nih.gov/research-topics/trends-statistics/overdose-death-rates

[45] WHO . Alcohol. World Health Organization. Published May 7 2022. Accessed January 18, 2023. https://www.who.int/news-room/fact-sheets/detail/alcohol.

[46] Berridge KC , Kringelbach ML . Pleasure systems in the brain. Neuron. 2015;86(3):646‐664. 25950633 10.1016/j.neuron.2015.02.018 PMC4425246

[47] Okun MS . Deep‐brain stimulation‐‐entering the era of human neural‐network modulation. N Engl J Med. 2014;371(15):1369‐1373. 25197963 10.1056/NEJMp1408779

[48] Müller UJ , Voges J , Steiner J , et al. Deep brain stimulation of the nucleus accumbens for the treatment of addiction. Ann NY Acad Sci. 2013;1282:119‐128. 23227826 10.1111/j.1749-6632.2012.06834.x

